# Supplementary material for: Achieving physical examination competence through optimizing hands-on practice cycles: a prospective cohort comparative study of medical students
Source: PeerJ. 2021 Dec 1;9:e12544. doi: 10.7717/peerj.12544 (PMC8643100; doi:10.7717/peerj.12544)
Supplement: Supplemental Information 2 [file peerj-09-12544-s002.docx]

**Table 1**. Demographics of the questionnaires

| Variables | Options | Samples (%) |
| --- | --- | --- |
| Instructor's version |  |  |
| Have you ever instructed a PE skill? | Yes | 200 (97.09) |
|  | No* | 6 (2.91) |
| How long have you been an instructor in PE training? | 1-5 years | 117 (58.50) |
|  | 6-10 years | 52 (26.00) |
|  | More than 10 years | 31 (15.50) |
| Student's version |  |  |
| What is your current stage? | Undergraduate | 159 (93.53) |
|  | Master student | 3 (1.76) |
|  | Doctoral student | 8 (4.71) |

* Instructors who said “no” for the question were excluded for further analysis.

**Table 2**. Suggestions about how to improve learning efficiency and outcomes of students in PE training course.

| Version | Suggestions | Samples (%) |
| --- | --- | --- |
| Instructor's version | More interaction with students | 74 (37.00) |
|  | Enrich teaching content | 47 (23.50) |
|  | Make students practice more | 29 (14.50) |
|  | Raise quality of instructors | 28 (14.00) |
|  | Add assessments in the end of classes | 13 (6.50) |
|  | Modify curriculum | 4 (2.00) |
|  | Do not know | 5 (2.50) |
| Student's version | Practice more in the practice session | 100 (58.82) |
|  | Enrich teaching content | 26 (15.29) |
|  | Modify curriculum | 11 (6.47) |
|  | Raise quality of instructors | 5 (2.94) |
|  | Add assessments in the end of classes | 5 (2.94) |
|  | Do not know | 5 (2.94) |

**Figure legends**

**Figure 1.** Flowchart of the questionnaire.

**Figure 2.** Results of the instructor questionnaires. A. What range do you want students to master the skill at the end of the course? B. What percentage of students do you think will meet your expectations? C. Do you know how many times students can practice on average in a PE training course? D. If your answer is “Yes” for the previous question, how many times did your students practice on average in the PE training course? E. How many practice cycles do you think would be appropriate for students to master PE skill in class? F. Have you considered or tried to increase the number of practice cycles for each student in the PE training course?

**Figure 3.** Results of the student questionnaires. A. How many hands-on practice cycles do you usually get in PE training class? B. Do you think you have enough practice chance? C. In the PE training course you have attended, did the instructors remind you to practice more? D. Do you consciously practice more cycles than planned in the PE training course without being reminded? E. How well do you master PE skill after the class? （range from 0 to 10: 0 represents no mastery of PE skill while 10 represents perfect mastery of PE skill）

**
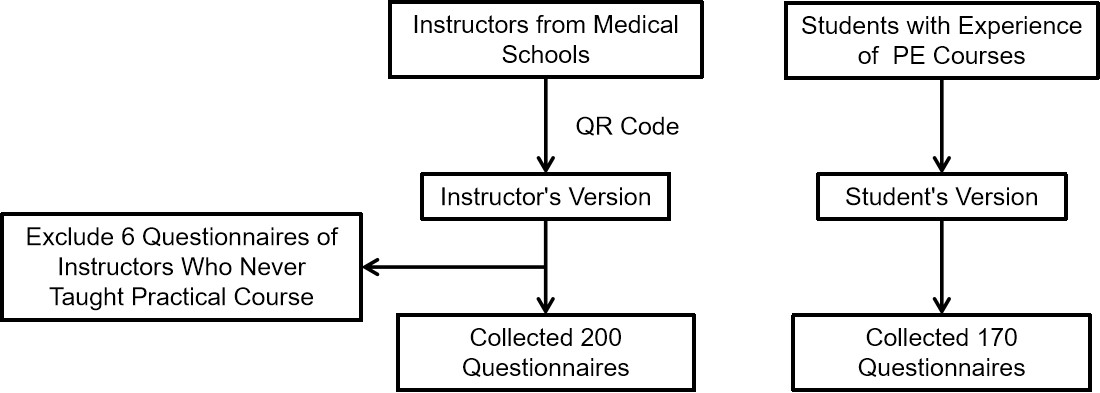
**

**Figure 1.** Flowchart of the questionnaire.


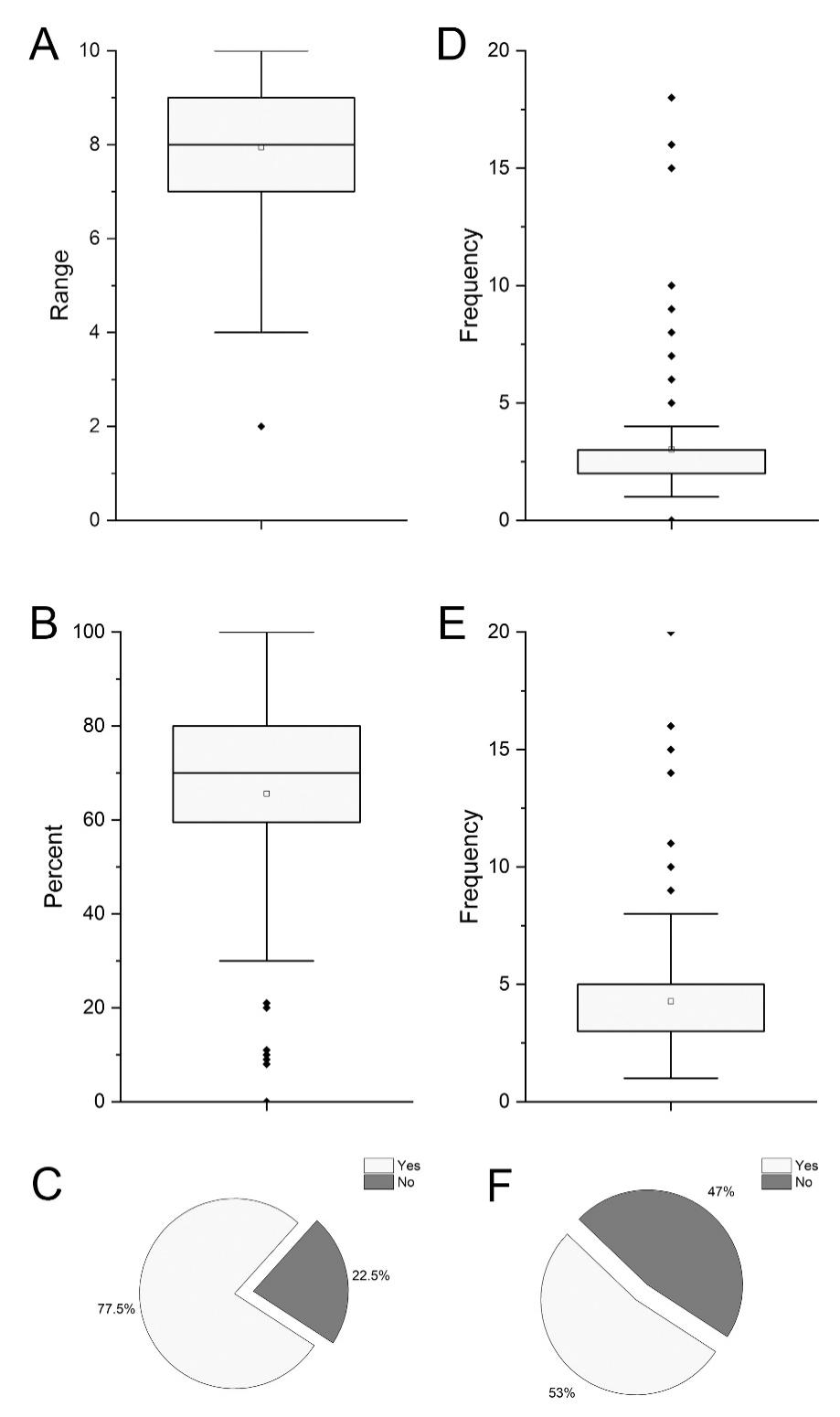


**Figure 2.** Results of the instructor questionnaires. A. What range do you want students to master the skill at the end of the course? B. What percentage of students do you think will meet your expectations? C. Do you know how many times students can practice on average in a PE training course? D. If your answer is “Yes” for the previous question, how many times did your students practice on average in the PE training course? E. How many practice cycles do you think would be appropriate for students to master PE skill in class? F. Have you considered or tried to increase the number of practice cycles for each student in the PE training course?


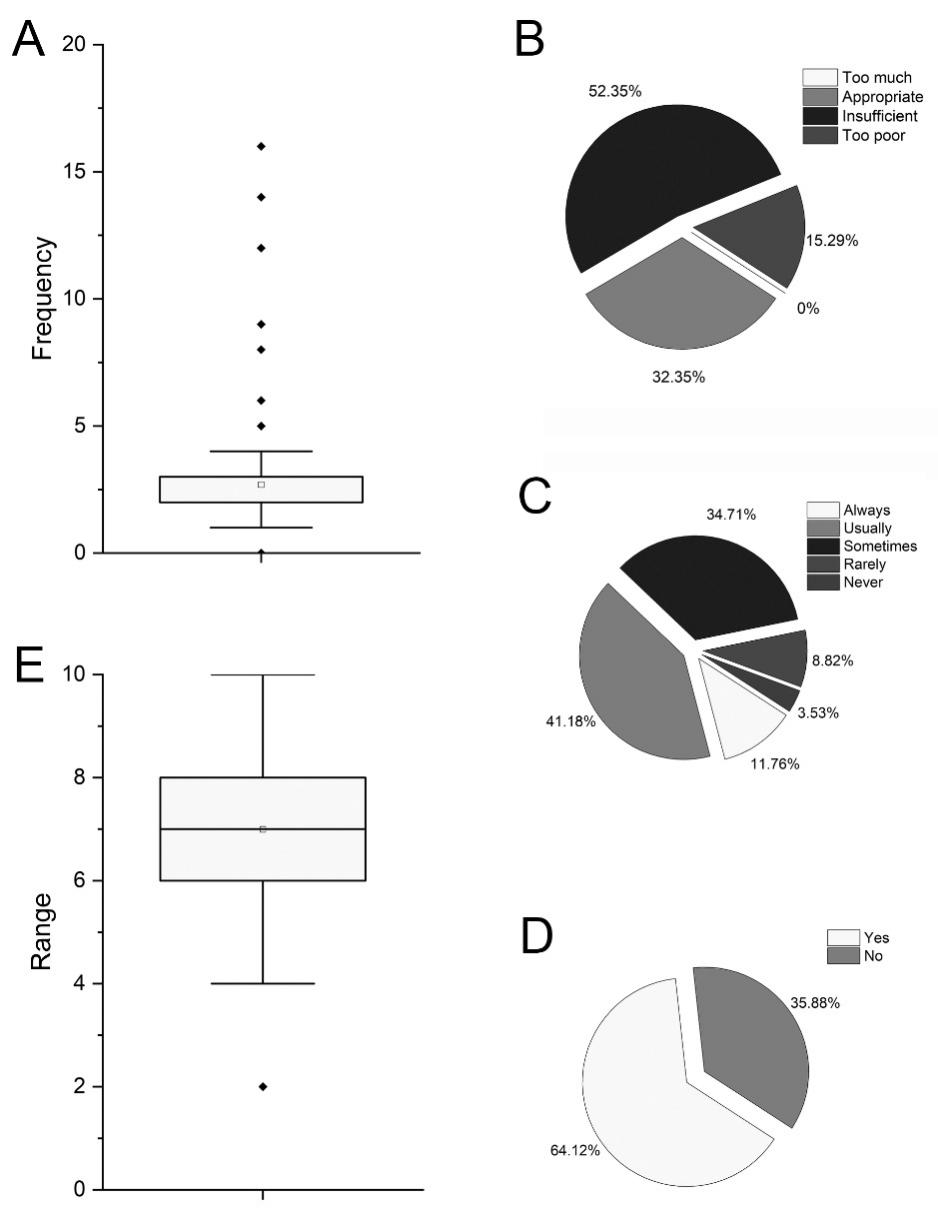


**Figure 3.** Results of the student questionnaires. A. How many hands-on practice cycles do you usually get in PE training class? B. Do you think you have enough practice chance? C. In the PE training course you have attended, did the instructors remind you to practice more? D. Do you consciously practice more cycles than planned in the PE training course without being reminded? E. How well do you master PE skill after the class? （range from 0 to 10: 0 represents no mastery of PE skill while 10 represents perfect mastery of PE skill）
